# European guidelines for constitutional cytogenomic analysis

**DOI:** 10.1038/s41431-018-0244-x

**Published:** 2018-10-01

**Authors:** Marisa Silva, Nicole de Leeuw, Kathy Mann, Heleen Schuring-Blom, Sian Morgan, Daniela Giardino, Katrina Rack, Ros Hastings

**Affiliations:** 1 Departamento de Genética Humana, Instituto Nacional de Saúde Dr. Ricardo Jorge, Lisboa, Portugal; 2 Department of Human Genetics, Nijmegen Donders Institute for Brain, Cognition and Behaviour, Radboud University Medical Center, Nijmegen, The Netherlands; 3 Genetics Department, Viapath Analytics, Guy’s Hospital, London, SE1 9RT UK; 4 Department of Genetics, University Medical Center Utrecht, Utrecht University, Utrecht, The Netherlands; 5 All Wales Genetics Laboratory, Institute of Medical Genetics, University Hospital of Wales, Cardiff, Wales UK; 6 Lab. Citogenetica Medica, Istituto Auxologico Italiano, Milano, Italy; 7 CEQAS/GenQA, John Radcliffe Hospital, Oxford University Hospitals NHS Trust, Oxford, OX3 9DU UK

**Keywords:** Genetics, Scientific community and society

## Abstract

With advancing technology and the consequent shift towards an increasing application of molecular genetic techniques (e.g., microarrays, next-generation sequencing) with the potential for higher resolution in specific contexts, as well as the application of combined testing strategies for the diagnosis of chromosomal disorders, it is crucial that cytogenetic/cytogenomic services keep up to date with technology and have documents that provide guidance in this constantly evolving scenario. These new guidelines therefore aim to provide an updated, practical and easily available document that will enable genetic laboratories to operate within acceptable standards and to maintain a quality service.

## Introduction

Chromosomal analysis has been a key tool in genetic analysis with conventional, as well as molecular, cytogenetics playing a crucial role over the years in many genomic disorders and in many laboratory settings. The overall application of these techniques by clinical genetics laboratories led to the need for harmonising cytogenetic practices and produce guidelines applicable throughout Europe. This was first achieved, in 2009, by the Permanent Working Group ‘Cytogenetics and Society’ European Cytogenetics Association and updated in 2012 [1]. Those documents have given the guidance on generic, as well as specific aspects of diagnostic guidelines and covered constitutional cytogenetics, acquired cytogenetics, microarrays and molecular-based techniques. However, given the advances in genetic technology and the shift towards molecular genetic techniques (e.g., microarrays, next-generation sequencing), as well as the application of combined strategies for the diagnosis of chromosomal disorders, it is essential that the cytogenetic/cytogenomic guidelines keep up to date. These new guidelines therefore aim to provide an updated, practical and easily available document that may be used by European Clinical Genetics laboratories. These guidelines take into account good laboratory practice documents, the existing external quality assessment schemes, accreditation procedures and protocols from different countries, as well as international policy documents (see Appendix A) and therefore, they apply unless overridden by national/federal laws, regulations and/or standards. The following standards should be considered as minimum acceptable criteria for constitutional chromosomal/cytogenomic analysis and therefore, any laboratory consistently operating below the minimum standard may be at risk of failing to maintain a quality service. Specific guidelines referring to cytogenomic analysis in acquired disorders are included elsewhere [2, 3].

This document is organised in two sections: general and specific guidelines. General guidelines address fundamental criteria to follow when providing cytogenomic testing regardless of the methodology applied. Specific guidelines supplementing these general guidelines include recommendations for each technique/methodology in particular. Both sections provide minimum acceptable criteria for sample preparation, analysis and reporting, as well as the limitations of the tests.

Cytogenomics is used herein as a general term that encompasses conventional, as well as molecular cytogenetics (fluorescence in situ hybridisation (FISH), microarrays) and molecular-based techniques. Specific designations are used when needed, otherwise the global term applies. Please note that non-invasive prenatal testing (NIPT) is not included in these guidelines as this is a screening test not a diagnostic test. Recommended practice guidelines regarding reporting NIPT results have recently been described by Deans et al. [4].

The use of terms such as ‘should’, ‘must’ or ‘essential’ are mandatory requirements, whereas the use of terms such as ‘may’ and ‘could’ are recommended, but not mandatory.

## General guidelines

This section addresses fundamental criteria to follow when providing diagnostic testing regardless of the methodology applied.

Service requirements regarding equipment, facilities, staff and diagnostic workload must comply with ISO15189 and/or ISO17025. All pre- and post-analytical procedures should also follow written protocols/standard operating procedures (SOP) in compliance with ISO15189 and/or ISO17025.

### Pre-analytical recommendations

Whenever a cytogenomic abnormality is suspected by the clinician as underlying a given patient’s condition/disorder a cytogenomic analysis should be considered. As with all genetic testing, informed consent for genetic testing should be given; whether a copy if this written consent is required by the laboratory will depend on National policy. Therefore, genetic counselling and communication, pre- as well as post-testing, is of utmost importance. Appropriate expert counselling may be provided by a medical doctor, nurse or a clinical laboratory specialist trained in the field of genetics. Patients must be informed about the scope, principle, resolution and limitations of the tests available for their specific context/situation. The possibility of incidental findings, (unrelated to the clinical question but nevertheless of importance for the individual’s health) and of uninformative or uncertain results should be addressed. Counselling is also vital after testing to aid the correct interpretation of the results and ensure the patient can truly make an informed decision.

Although local policies might differ throughout countries, the following list is given as guidance to delineate the type of referral indications and of the expected scope of a cytogenomics service:

#### Main clinical indications for prenatal diagnosis

- Abnormal foetal ultrasound;

- High-risk maternal serum screening/NIPT result indicating an increased risk of a chromosomally abnormal foetus;

- Parental chromosome rearrangement, mosaicism or previous aneuploidy;

- Previous livebirth/stillbirth with a chromosome abnormality;

- Possible foetal mosaicism detected by prior prenatal study;

- Familial monogenic disorder (i.e., CF, Noonan syndrome, etc).

#### Main clinical indications for postnatal diagnosis

- Abnormal clinical phenotype;

- Multiple congenital abnormalities;

- Clinically significant abnormal growth – short stature, excessive growth, microcephaly, macrocephaly;

- Primary or secondary amenorrhoea or ovarian insufficiency;

- Ambiguous genitalia;

- Infertility of unknown aetiology;

- Sperm abnormalities – azoospermia or severe oligospermia;

- A malformed foetus or stillbirth of unknown aetiology;

- Third and subsequent miscarriage(s): products of conception;

- Significant familial history of chromosome rearrangements;

- Significant familial history of intellectual disability (ID) of possible chromosomal origin where it is not possible to study the affected individual;

- Carriership for an X-linked recessive disorder in a female.

#### Other postnatal indications may include

- Parental follow-up of a chromosomal abnormality or variant detected in a pre- or postnatal foetal/child’s sample;

- Delineation of an abnormal cytogenomic result;

- Refractory epilepsy for mosaicism exclusion.

When deciding which methodology to apply, the specific referral indication and the advantages and limitations of each technique must be taken into account (e.g., resolution, reporting time) (Table 1).

**Table 1:** Methods used in cytogenomic analysis, their resolution and limitations

Method	Resolution	Limitations
Karyotyping	5–10 Mb	Cannot detect: small rearrangements below the resolution; nucleotide variants; mosaicism < 10%^a^; UPD^b^
FISH	~100 kb	Limited to the probes used (targeted analysis); Cannot detect: nucleotide variants; mosaicism < 10%^c^; UPD
Array-based techniques chromosomal microarray SNP-based array	~20–200 kb	Cannot detect: balanced rearrangement; mosaicism < 10%^d^; nucleotide variants; the nature of a structural aberration; independent cell lines; heterochromatic markers; triploidy (exception SNP array); UPD (exception SNP array)
CNV detection in whole-exome sequencing	100 bp (exonic regions) – ~150 kb (genome wide)^e^	Cannot detect: balanced rearrangement; mosaicism < 18%^f^; the nature of the structural aberration; independent clones/cell lines

^a^Hsu and Benn [7]; Hook [36]

^b^Uniparental disomy

^c^Wiktor and van Dyke [37]; Ballif et al. [38]; Mascarello et al. [39]

^d^Vermeesch et al. [23]; Ballif et al. [38]

^e^Pfundt et al. [32]

^f^Pagnamenta et al. [40]

### Sample preparation

Depending on the referral reason, the tissue types and sample preparation may vary. However, there are general recommendations that should be considered when preparing samples for cytogenomic analysis.

Cultures set-up for prenatal samples should be performed in duplicate or independently, where possible. For chorionic villus samples (CVSs) it is recommended to establish and analyse long-term cultures (LTCs, cytotrophoblast and mesenchymal cells), even if short-term cultures (STCs; cytotrophoblast cells) are in place, to allow for mosaicism exclusion when needed (see below). In embryo development, the mesenchymal cells more closely represent the foetal genotype than cytotrophoblast cells and therefore long-term CVS cultures are more likely to represent the foetal karyotype than short-term cells/cultures [5, 6]. Back up cultures of prenatal samples should be kept until the final report is written, and when practicable, the possibility of freezing viable cells, as well as fixed cell pellets should be available for cases that may require further cytogenomic or molecular analysis. For postnatal analysis, blood samples may be stored in the refrigerator for up to 5 days and a second culture initiated if required.

DNA extraction methods, should either be automated or require minimal tube–tube transfers and produce DNA that is reliable for use in the techniques applied thereafter if possible. There must be internal criteria for ascertaining the DNA quality and quantity, as well as the minimum threshold for suitability for a given test. Precautions must be in place to minimise PCR product contamination therefore a separation of pre- and post-PCR areas is required. Controls must be included in each PCR set-up to identify DNA or PCR product contamination.

The most critical issues regarding sample preparation involve ensuring, as much as possible, that the samples are correctly identified (i.e., sample verification) and suitable for the technique used, as well as minimising the possibility of misdiagnosis. For prenatal samples, a minimum of two cultures should be set-up and analysed, although for follow-up of an abnormal rapid result only a single culture is required, providing the results are concordant. Maternal cell contamination (MCC) and confined placental mosaicism (CPM), in the case of CVS, may greatly increase the possibility of misdiagnosis. According to Hsu and Benn [7], three cultures should be analysed for mosaicism exclusion. MCC may be due to the presence of blood in amniotic fluid (AF) samples or of maternal decidua in CVS, for instance. In the latter case, it is essential that the CVS is thoroughly cleaned and maternal decidua removed prior to DNA preparation and/or culture set-up. Nevertheless, whenever the origin of the prenatal sample, which is female is in doubt, genotype analysis of a maternal (blood) sample should be performed. Specifically, genotype analysis is recommended for poor quality CVS where placental villi cannot be confidently identified and for AF pellets exhibiting a significant* amount of blood staining (*as determined by the laboratory).

To avoid misdiagnosis due to CPM, it is recommended that heterogeneous cell populations are analysed. This may be done using several villi, instead of just one, for the preparation of the DNA pools and/or cultures. It is also recommended that the same pool of chopped or digested villi are used for molecular-based analysis and culture set-up to minimise discrepant results [8].

A checking procedure should be in place for all critical transfer steps (i.e., setting up or when transferring details of identity).

### Analysis

Laboratories must ensure that the staff have the skills and expertise, as well as collaborations and supervisory arrangements, to perform the tests and correctly interpret the findings. Appropriate training for the types of samples to be analysed must be ensured and written criteria (SOP) for classification of observations and interpretation of results should be in place.

All analyses should be performed by qualified professionals trained in Clinical Laboratory Genetics and be independently checked by a second trained member of staff, who could be a technician or a clinical laboratory geneticist, the latter also being responsible for signing out the report. An independent check by a ‘blind’ analysis is recommended as this prevents any bias.

According to the methodology used, specific requirements for the analysis are recommended (see section Specific guidelines).

### Reporting

The reporting of results should be standardised as far as possible, unambiguous, informative and clear to read by the non-specialist in order to be understood by the recipient/clinician. A summary banner should be given that includes all the relevant results. All reports should include an interpretation of the results and answer the clinical referral/question. Long reports should be avoided as this detracts from the clarity of the results. Methodology and limitations of the test should not take prominence in a report as they can detract from the results and interpretation.

Reporting times should be kept as short as possible and should take into account the reason for referral and level of urgency (see Table 2).

**Table 2:** Recommended reporting times (calendar days) for 90% of the referrals

Prenatal aneuploidy testing by FISH /QF-PCR/MLPA	4 days
Amniotic fluid and CVS analysis on cultured or uncultured cells by karyotyping and/or genome-wide array analysis	14 days
Lymphocyte cultures	28 days
Products of conception/foetal skin (where pregnancy is not ongoing)	28 days
Urgent^a^ lymphocyte, cord blood cultures	7 days
Postnatal array analysis	10 days (urgent) 28 days (other)

These apply in the absence of specific National Guidelines

^a^URGENT – referrals where the result will have immediate implications for patient management

The results should be given using the most recent International System for Human Cytogenomic Nomenclature (ISCN) [9], where practicable. Human Genome Variation Society (HGVS) nomenclature (http://www.HGVS.org/varnomen) should be used when applicable. HUGO Gene Nomenclature Committee (HGNC) gene names should be used in ISCN, where applicable. The results can be tabulated if the result is complex. Single-cell anomalies and heteromorphisms should not be included in the ISCN in a diagnostic report.

All reports, regardless of the methodology used, should comply with ISO15189 and include the following information [10]:

- Reason for referral/clinical indication for the test;

- Date of sampling, date of receipt and the date of report;

- Patient identification using two different identifiers (IDs), i.e., full name and birth date; Twins IDs must be clearly distinct;

- Gender of the patient (postnatal referrals – essential for sex chromosome referrals);

- Unique sample identifier;

- Name of referring clinician and hospital;

- Laboratory identification;

- Tissue examined (i.e., DNA isolated from cultured fibroblasts);

- A clear summary of the results (ISCN, HGVS or as a summary statement) (i.e., not hidden within the text);

- A written description of the results including the sex of the patient (for prenatal samples stating the genetic sex is optional);

- A written interpretation of the results (understandable by a non-specialist);

- Relating the result to the referral reason and/or answering the clinical question;

- Specification of which kit/probe (QF-PCR/MLPA/FISH) or array platform is used and stating the respective test limitations;

- Patient identification should be present on each page;

- Name and signature of one or two authorised persons, which may include the Section Head/Clinical Laboratory Geneticist. The signature may be generated electronically or manually;

- Pagination (i.e., page 1 of 1 or page 1 of 2).

In addition to the information stated above, reports of abnormal results should also include, when appropriate, the following:

- A clear written description of the abnormality;

- Whether the result is consistent with the clinical findings, and/or an indication of the expected phenotype/syndrome (e.g., ‘consistent with ___ syndrome’, or ‘associated with ___ syndrome’, or ‘indicative of ___ syndrome’);

- In case of intragenic imbalances (detected by array, MLPA and/or CNV in exome data), the HGNC gene symbol should be mentioned and it is recommended to also specify the reference transcript (representing either the longest known transcript and/or the most clinically relevant transcript);

- Any follow-up tests required to confirm the results and/or suspected diagnosis, preferably stating, which samples (i.e., heparin or EDTA blood, buccal swab, etc.) are required for these specific tests;

- Assessment of recurrence, when appropriate;

- Prenatal diagnosis in future pregnancies, if appropriate;

- Recommendation for genetic counselling;

- Comment stating, where applicable to the disorder, ‘Please ensure the karyotype record appears in the child’s own notes’ (after delivery for prenatal samples);

- Request for follow-up of family members at risk of the abnormality, starting with closest available relatives;

- Minimum threshold used for reporting ‘abnormal’ results.

The presence of MCC or pseudomosaicism should always be noted on the laboratory record but should not be commented upon on the final report unless relevant for result interpretation. For mosaic results found in CVS, CPM should be discussed in the report, if applicable. AF mosaic reports should consider that the proportions of the abnormal and normal cell lines may be different in foetal tissue(s). The need for pre- or postnatal follow-up samples and/or parental samples should always be made clear, where appropriate.

## Specific guidelines

The following recommendations supplement the general guidelines (see section General guidelines) and give more specific guidance on constitutional cytogenomic analysis performed using the techniques currently available.

### Karyotyping

All karyotyping should be carried out using a banding technique, with G-banding being the most widely used technique. The laboratory has to ensure that whichever banding technique is used it has to achieve the required banding quality (see Table 3). ISCN (2016) [9] defines five levels of banding and Table 3 gives the minimum quality levels that must be met to report a ‘normal’ result.

**Table 3:** Minimum G-banding quality according to the reason for referral

Reason for referral	Minimum G-banding quality (QAS) ^a^
Confirmation of aneuploidy	QAS 2^a^ ⇔ < 300 bphs
Exclusion of known large structural rearrangements	QAS 3 ⇔ 300 bphs
Identification and exclusion of small expected structural rearrangements; routine prenatal specimen preparations	QAS 4 ⇔ 400 bphs^b^
Prenatal specimen abnormal ultrasound referrals (in the absence of array-based analysis)	QAS 5 ⇔ 550 bphs^b^
Routine postnatal specimen preparations	QAS 6 ⇔ 550 bphs^b^

*bphs* bands per haploid set

^a^QAS score – see ACC Professional guidelines for clinical cytogenetics v1.04 (2007; http://www.acgs.uk.com/media/765607/acc_general_bp_mar2007_1.04.pdf)

^b^SNP-based array or other molecular techniques/SNP-based array or other molecular techniques may be more applicable for some of these referral categories

Full analysis must be completed and checked by the supervisor ensuring the minimum quality levels appropriate for the referral reason are met. Where it is not possible to achieve the minimum quality for the referral reason, and no abnormality is detected, the report should be suitably qualified while not encouraging a repeat invasive procedure when this is not clinically justified. Targeted analysis may be appropriate to investigate abnormalities identified by another method, for example, to establish the chromosome abnormality and recurrence risk for prenatal cases identified as having a chromosome aneuploidy by QF-PCR.

The laboratory should have written protocols for analysis. Incomplete/broken metaphases should not be included in the analysis. Metaphase analysis must involve a comparison of every set of homologues (including sex chromosomes), band by band. If one homologue pair is involved in an overlap with another chromosome, the pair of homologues should be independently scored in another metaphase to ensure there is no structural rearrangement. Therefore, additional cells have to be counted and analysed to complete the analysis. For guidance on the number of metaphases that should be analysed for each type of tissue see Table 4. In general, a minimum of two cells must be fully analysed (at the minimum quality for the respective referral reason), although in practice more metaphases are counted and analysed to clear any crossovers. If mosaicism is clinically indicated or suspected, an extended analysis and/or cell count must be performed (see Table 4 for number of cells and also the latest ISCN for definition of a clonal abnormality). Laboratories may choose to analyse or count more cells routinely than the minimum given in Table 4 to exclude mosaicism for all referrals – not just where suspected from the referral information. Internal reports should include the number of cells analysed and counted, with relevant details provided.

**Table 4:** Minimum number of metaphases to be analysed according to tissue type

Sample	Referral/result	Minimum analysed^a^	Additional cells counted^b^
Prenatal	Routine	2 (2 cultures)^c^	0^d^
Postnatal	Routine	2^e^	0
Pre- and postnatal	Mosaicism exclusion or single-cell anomaly detection	2	28^f^

^a^Analysed (banded metaphases where every set of homologues without any crossovers are evaluated in their entirety). (In practice more metaphase are analysed to clear any crossovers – see section Karyotyping)

^b^Counted (metaphases where the number of centric chromosomes and/or the presence/absence of a specific cytogenetic feature is evaluated)

^c^QF-PCR and one culture in case of aneuploidy testing

^d^Extra cells may be counted to exclude mosaicism or a single-cell anomaly

^e^ACC Professional guidelines for clinical cytogenetics v1.04 (2007; http://www.acgs.uk.com/media/765607/acc_general_bp_mar2007_1.04.pdf) and Hastings et al. [1]

^f^Hsu and Benn [7] and Hook [36]

A report can be issued upon detection of an imbalance in the proband whether or not the sample was referred individually or with parental blood samples. Comments on the clinical significance may be made if a phenotypic association is supported in the published literature, otherwise it is appropriate to report the anomaly as having unknown significance. Polymorphic variants that have been previously reported as harmless should be excluded from the report (to avoid confusion for the non-specialist) and only documented in the patient’s laboratory record [5]. Cytogenetically visible polymorphic variants include variants involving heterochromatin (variant size), satellite size, pericentric inversions (heterochromatic or euchromatic regions) [e.g., 1qh+/qh-, 9qh+/qh-, 16qh+/qh-, acrocentric p+ or p-, Yqh+/qh-, inv(9)(p11q13), inv(2)(p11.2q13)] and also euchromatic variants (e.g., located on 4p16, 8p23.1, 9p12, 9q13-q21.12, 15q11.2, 16p11.2) [11].

Mosaicism or pseudomosaicism should also be excluded from the report if it is likely to be a cultural artefact after appropriate work-up (see Gardner and  Amor) [5]. Distinction between a cultural artefact and true mosaic aberration is not always easy, so application of general rules together with consideration of the clinical referral need to be kept in mind when reaching a decision (please refer to ISCN [9], EUCROMIC study [12] or the ACC collaborative study [13]). If XX/XY mosaicism (in prenatal samples) is >10% or is level III, further investigations should be done (e.g., quantitative fluorescence-polymerase chain reaction (QF-PCR)) before reporting.

### Chromosome instability syndromes

As this type of syndromes is rare and challenging both in terms of analysis and interpretation, it is recommended that these cases are referred to experienced/reference labs with proven expertise. Classic chromosome breakage disorders include Ataxia telangiectasia (AT), Bloom syndrome, Fanconi anaemia, Nijmegen breakage syndrome (NBS), Roberts’ syndrome, Immunodeficiency–Centromeric instability–Facial anomalies (ICF) syndrome and Mosaic Variegated Aneuploidy (MVA) [5, 14]. Other syndromes involving defective DNA replication/repair (e.g., Cockayne syndrome and Xeroderma pigmentosum) are not amenable to cytogenetic methods of confirmation. Although cytogenetic studies are often the first step in making a diagnosis, continuing research has led to identification and cloning of putative disease genes. Therefore it is recommended that molecular testing is considered instead of/or to supplement cytogenetic analysis, in particular now that targeted exome sequence analysis is available in some diagnostic laboratories.

When analysing breakage syndrome referrals, it is essential that sufficient numbers of metaphases are examined in order to ensure that any chromosomal damage detected is significant. Clastogen studies must only be undertaken with appropriate negative matched control samples and, if available, positive matched control samples. All control and test samples should be processed, cultured and harvested in parallel. Controls should be appropriately matched (e.g., sex, age etc.). The patient and control samples should be analysed blindly.

For Bloom syndrome, it is recommended that 20 harlequin banded metaphases are examined as some patients have a population of cells with a normal sister chromatid exchange (SCE) frequency. The laboratory should have an internal record of the SCE frequencies found when the same methods are applied to a range of normal control samples. As the *BLM* gene has been identified in association with Bloom syndrome, molecular testing to confirm the cytogenetic result is now possible.

Analysis of at least 50, but preferably 100 metaphases, is recommended for Fanconi anaemia to exclude the possibility of a somatic variant, which is common in these patients. To ensure the efficacy of the clastogen used SCE levels should be analysed in negative cases. The mean breakage per aberrant cell and the mean breakage per normal cell should be calculated. Referral to molecular analysis of complementation groups, or targeted exome sequencing is also recommended for abnormal results.

For AT and NBS, the aberration frequency in irradiated cultures should be calculated by scoring 50–100 metaphases. As some patients display an intermediate response to irradiation, screening of 50 banded metaphases for rearrangements, involving the T-cell antigen receptor loci on chromosomes 7 and 14, should also be carried out. As the *ATM* and *NBN* genes have been cloned (and associated with AT and NBS, respectively) molecular analysis for confirmatory purposes should be suggested for patients with positive cytogenetic and/or radio sensitivity assays.

Roberts’ syndrome referrals require scoring of 50 block (Leishman/Giemsa stained) or C-banded metaphases for centromere puffing and tramline chromosomes. Fifty banded metaphases should be counted for evidence of aneuploidy.

Fifty banded metaphases should also be scored in ICF syndrome cases for anomalies of the heterochromatic regions of chromosomes 1, 9 and 16 and for multi-branched configurations.

MVA is characterised by mosaic aneuploidies, predominantly trisomies and monosomies, involving multiple different chromosomes together with normal cells. Sometimes these are single-cell anomalies but a proportion of metaphases with aneuploidy > 25% is required to define the disorder (OMIM #257300).

### Fluorescence in situ hybridisation

FISH can be applied to interphases and/or metaphases, for a variety of clinical applications. Usually it addresses a specific clinical question and therefore it is important that the clinical reason for testing is provided on the referral form. Single probe or multiple chromosome probes can provide reliable results in different clinical situations (Tables 5 and 6). Whole chromosome paints (wcp) have a limited application and are only applicable for metaphase FISH analysis to identify large imbalances or rearrangements ( > 5–10 Mb). In most instances, the clinical laboratory geneticist will decide whether FISH or another testing method is more appropriate depending on the referral reason. Interphase FISH could be used as a method of choice in instances where rapid test results are required, for example, to detect trisomy 21 or sex chromosome constitution in newborns. Interphase FISH is also used for aneuploidy testing (trisomies 13, 18, 21 and sex chromosome aneuploidies) as an alternative method to QF-PCR or multiplex ligation-dependent probe amplification (MLPA) in prenatal diagnosis or for routine investigation of pregnancy losses.

**Table 5:** Applications of FISH analysis

Type of FISH analysis	Applications of FISH analysis
Rapid prenatal FISH	High risk of chromosome aneuploidy or recurrent microdeletion (e.g., abnormal ultrasound); late gestation referral.
	Evaluation/characterisation of
Interphase FISH	Numerical abnormalities; Duplications; Deletions; Sex chromosome constitution; Mosaicism; Gene amplification.
Metaphase FISH	Marker chromosome; Unknown material attached to a chromosome; Rearranged chromosome(s); Suspected gain or loss of a chromosome segment; Mosaicism.

**Table 6:** Minimum criteria for analysing FISH results according with probe type

Probe type	Analysis	Additional comments
Locus-specific identifier (LSI) probes	5 metaphases	Score to confirm or exclude an abnormality (e.g., in case of suspected microdeletion syndrome or identification of a marker chromosome)
Multiprobe analysis	3 metaphases	Per probe. Scored to confirm a normal signal pattern. Confirmation is advisable for abnormal signal patterns if no control probe is present
Interphase analysis for aneuploidy testing	≥50 cells	For each probe set
Interphase analysis to detect mosaicism	≥100 cells	For each probe set

Metaphase FISH, on the other hand, is a method of choice for the detection of submicroscopic balanced rearrangements and can be used for chromosome imbalances when targeted proband/family follow-up investigations of abnormalities detected by array analysis are required.

FISH can be performed pre- or postnatally. Postnatally, FISH can be used on virtually every tissue (e.g., lymphocytes obtained after blood withdrawal, skin tissue, urine specimens, buccal swabs, tissue derived from products of conception, etc.). Prenatally, FISH can be performed on either direct slides from uncultured (interphase) or cultured amniocytes, as well as on CVS slides of STC (trophoblast cells) or LTC (mesenchymal cells). Blood staining in an AF sample should be noted as these samples may contain MCC (see section Quantitative fluorescence-polymerase chain reaction). In case of suspected mosaicism or to rule out CPM, FISH on STC, as well as LTC slides can be performed.

Individual FISH probes that are used repeatedly in routine diagnostics should be validated to ascertain the upper (with known abnormal sample(s)) and lower thresholds (with normal sample(s)) for reporting a normal or an abnormal result [15]. Sufficient numbers of metaphases, or nuclei from cultured or uncultured cells must be analysed depending on the probe type to ensure the statistical validity of the result. Signals must be scored (Table 6) by two independent analysts or may be confirmed by another technique. There may be variation in probe signals both between slides (depending on age, quality, etc. of metaphase spreads) and within a slide. Where a deletion or a rearrangement is suspected, adequate hybridisation efficiency can be confirmed either by the presence of a signal on the normal chromosome or if a control probe is provided in the probe mix. Depending on the sensitivity and specificity of the probe and on the number of cells scored, the possibility of mosaicism should be considered, and comments made where appropriate (Table 5). FISH analysis may be the most appropriate method of confirming suspected mosaicism if a suitable probe is available to assess levels of mosaicism or chimerism of different cell lines. Low-level mosaicism (10%) should be interpreted with caution as this is within the limitations of the assay. In some instances, more than one tissue type should be investigated (e.g., if Pallister–Killian syndrome or trisomy 8 mosaicism is suspected). The laboratory geneticist may decide that fewer cells than indicated are analysed in cases where screening for a specific abnormality is the main indication for the study (e.g., checking for a known familial abnormality) or when an abnormality is detected but there are no more cells available. The test should be repeated when hybridisation results are not optimal. Most FISH results should be accompanied by a karyotype analysis, except when performed as follow-up test after array analysis or when discrepancies are found between the findings and the reason of referral.

FISH results should be related to the karyotyping or array results, when available, and can be reported together with a standard cytogenetic observation or array result (and combining both in the ISCN), or as a stand-alone test result. Normal FISH results should be reported within the text but do not need to be included in the ISCN. Abnormal FISH results should always be reported using the latest ISCN nomenclature and with the designation of the clone name (preferentially) or the locus designation (accession number, gene name or D-number). The probe manufacturer should also be provided. Internal reports should include the number of cells scored and the hybridisation details. Contiguous probes (contigs) may be reported following ISCN, i.e, reporting all the contigs separated by single slant lines or if only one contig probe for a specific chromosome is given in the ISCN the full contig position should be described in the report. Complex FISH results can be tabulated or given as a summary result instead of using ISCN. Mosaic findings should be related to the referral indication, where appropriate.

It should be made clear that FISH analysis does not substitute a complete cytogenomic analysis, provides information solely on the locus of the probe used and its limitations arise from the limitations of those probes (e.g., whether the probe localisation is within or flanking the gene, cross hybridisation with other positions in the genome). Furthermore, normal results from highly repetitive sequence probe analysis should be interpreted carefully due to the possibility of individuals with rare polymorphisms involving those sequences.

Care needs to be taken in interpreting interphase FISH results. The signal in interphase cells can be variable, therefore > 50 cells, must be examined. It should be noted that this analysis is only able to detect a subset of abnormalities and may be incomplete or misleading in the absence of a karyotype analysis (e.g., der(21;21) or a sex chromosome rearrangement).

Caution should be taken when interpreting rearrangements using wcp probes results, and it should be performed in conjunction with banding studies or after an abnormal array result. It should be noted that the resolution of chromosome paint probes is at best 5 Mb. Small rearrangements may not be detected because wcp probes may not be uniformly dispersed across the full length of the target chromosome. Chromosome paints cannot be used to ascertain precise breakpoints.

In the case of single target probe results, i.e., locus-specific identifier (LSI), the number of cells scored needs to be in accordance with the sensitivity and specificity of the probe used for analysis – usually five metaphases is adequate. Microduplications may not be definitely identified by FISH analysis; in cases where a microduplication is suspected, confirmation by alternative methodologies (e.g., molecular analysis) is required.

### Quantitative fluorescence-polymerase chain reaction

QF-PCR analysis of microsatellite markers is widely used for the detection of common aneuploidies in prenatal, foetal tissue and new born blood samples. The approach has significant economy of scale, detects triploidy and mosaicism and genotypes the sample, allowing for identification of MCC, twin pregnancies and chimeras. For prenatal samples, it is recommended that chromosomes 13, 18 and 21 are always tested. If the sex chromosomes are not routinely tested, it is recommended that these are included for samples where ultrasound anomalies are suggestive of monosomy X (Turner syndrome). QF-PCR analysis is a diagnostic, targeted test and does not require confirmation by another approach. However, karyotype analysis should be undertaken following identification of aneuploidy to identify structural rearrangements. Where these are identified or where karyotype analysis of the original material is not possible, karyotype analysis of parental samples is indicated to assess the recurrence risk. QF-PCR analysis may be used as a stand-alone test for samples that are not at risk of other chromosome abnormalities. For prenatal samples referred with ultrasound abnormalities and a normal QF-PCR result, subsequent array analysis is recommended.

It is recommended that tri/tetra/penta/hexanucleotide repeat markers are used as these have fewer stutter peaks. Dinucleotide repeat markers are acceptable if stutter peaks are included in the analysis. Sufficient markers (at least four markers per chromosome) should be included in QF-PCR assays to minimise uninformative results and reporting delays. Assays should be designed to cover the length of the chromosome and to avoid clustering of markers. New markers that have not been previously reported for QF-PCR aneuploidy diagnosis should be validated by testing a minimum of 100 chromosomes (the equivalent to 50 unrelated DNA samples) to ensure they are not located in a common copy number variant (CNV). Unpublished primers should be checked and validated to ensure the primers sequences are not affected by common Single nucleotide polymorphisms (SNPs). Aneuploid samples should be included in all validations to ensure the correct chromosome location. It is recommended that home-made kits are batch tested using at least a trisomy and a normal DNA sample to ensure consistent assay quality.

If the PCR cycle number is changed, the use of an appropriate trisomy control is recommended to ensure that biallelic ratios are maintained. Alleles must be clearly separated. Either peak height or peak area ratios may be used, if validated. It is recommended that both the electrophoretogram and allele peak ratios are analysed to identify low-level cell lines. To ensure the quality of the data, both minimum and maximum peak heights should be established. In line with current practice, it is recommended that allele peak ratios are calculated by dividing the peak height or area of the shorter length allele by that of the longer length allele. Preferential amplification of the shorter length allele is a feature of QF-PCR and the normal and abnormal biallelic ratio ranges reflect this. Although there is a requirement for testing laboratories to validate/verify the biallelic ratio bins, normal ratios are generally between 0.8 and 1.4, whereas those representing trisomy are < 0.65 or > 1.8. Inconclusive ranges lie between the abnormal and normal bins. For non-polymorphic markers that are not subject to preferential amplification, such as sex chromosome sequences *TAF9L* and *AMELOGENIN*, a narrower allele range may be appropriate. It is recommended that allele ratios that indicate the different sex chromosome aneuploidies are determined.

To interpret a result as normal at least two informative marker results consistent with a normal biallelic pattern are required per chromosome, with all other markers uninformative. However, it is acceptable to report a single-marker result that has a normal biallelic pattern and all other markers uninformative as consistent with a normal chromosome complement, provided the report states that the result is based on a single-marker result and that this result must be confirmed. Abnormal polymorphic marker results either exhibit three different alleles in a 1:1:1 ratio or biallelic 1:2 or 2:1 ratios (see above). To interpret a result as abnormal (trisomy), at least two informative marker results should be consistent with trisomy with all the other markers uninformative. It is unacceptable to interpret a single-marker result as abnormal as this may represent a rare CNV or other polymorphism (see below).

To identify monosomy X, one or more paralogous markers (located on the X chromosome and an autosome, e.g., *TAF9L*) must be used in addition to polymorphic X chromosome counting markers and at least two Y chromosome-specific sequences. In the absence of paralogous markers, definitive diagnosis of monosomy or a deletion is not possible.

Where more than one sample is processed, it is recommended that sample identity of abnormal results is confirmed prior to reporting. This may be done by repeat PCR of the DNA (where samples are prepared in isolation), re-extraction and re-testing of the sample, or maternal genotype analysis.

For heavily blood-stained samples, sexing is recommended and if the sample is female, maternal genotype analysis should be performed to establish the origin of the genotype. The aneuploid status of samples exhibiting significant MCC, characterised by inconclusive allele ratios and/or the maternal genotype present at a high level, should not be determined. In these cases, the foetal genotype, must not be stated or reported. CVS should be expertly dissected to minimise MCC. Up to 2% of AF samples will exhibit significant MCC and are usually blood stained. In these cases, a QF-PCR result from uncultured fluid will not be available and analysis of cultured cells is recommended. It is recommended that an uninformative or ‘unsuitable sample’ report is issued.

When reporting a trisomy QF-PCR result, the name and location of informative markers should be listed to define the trisomic region. For monosomy X results, the name and location of paralagous markers should be given. The laboratory should have written procedures for reporting other sex chromosome abnormalities, e.g., XXX, XXY, XYY.

If a meiotic non-disjunction is indicated by the results this may be reported. If no triallelic results are observed, the increased risk of a mitotic non-disjunction event may be reported. Mosaic findings should relate to the referral indication where appropriate. Expert analysis can detect second cell lines to a level of at least 20%, however, mosaicism at lower levels may not be detected.

Where normal QF-PCR results are found in samples referred with a high-risk NIPT result, it is recommended that non-concordance of the QF-PCR with the NIPT result is highlighted and a statement that the QF-PCR diagnostic result supersedes the NIPT screening result should be included in the report. In the case of AF samples with no ultrasound anomalies, further analysis is not required. For CVS samples, analysis of mesoderm (digested villi) is recommended. In cases where CPM is a likely cause of the discrepant result, ultrasound monitoring should be recommended on the report.

The interpretation of QF-PCR results may be confused by the presence of polymorphisms, such as primer site polymorphisms (PSPs), CNV duplications or somatic microsatellite variation (mutations) (SMMs).

- PSPs, e.g., SNPs within the primer sequences, may cause reduced allele amplification and give an abnormal or inconclusive result. Lowering the annealing temperature of the PCR reaction usually facilitates primer hybridisation and allele amplification, and characterises a PSP. However, in these cases the marker should not be used for interpretation as amplification may not be complete.
- Informative markers within a CNV duplication will give an abnormal result at that locus, therefore single-marker abnormal results must not be reported as consistent with whole chromosome trisomy. If a single abnormal marker result is flanked by normal markers and has been previously reported in normal individuals then it does not have to be reported. However, if the abnormal marker is the most distal or proximal or has not been reported in a normal individual then further studies are recommended prior to interpretation. Parental studies or array analysis may provide further information.
- SMMs [16] constitute rare events that present either as a skewed allele ratio or three alleles in an A + B = C pattern. If a single marker gives a characteristic SMM pattern or inconclusive biallelic ratio, this does not have to be reported. Analysis of cultured cells may assist interpretation as the proportion of cells with the novel allele is likely to change.

If both normal and abnormal marker results are observed for a single chromosome, it is recommended that further investigations are carried out such as lowering the PCR annealing temperature, testing an additional aliquot/cultured cells, parental studies and/or array studies. Such a result may represent a polymorphism of no clinical significance or partial chromosome imbalance. If the result is not likely due to a SNP, CNV or SMM then it should be reported.

Finally, QF-PCR cannot detect any changes that lie outside the target sequence of the markers and will not detect balanced rearrangements. As with all targeted tests, even when no aberrations are detected, the possibility remains that biological changes in that gene or chromosomal region do exist but remain undetected.

### MLPA-based techniques

MLPA is a semiquantitative PCR technique that relies on ligation of specific probes on adjacent DNA sequences and multiplex amplification of these probes [17, 18]. This methodology allows for the detection of copy number (CN) changes (deletions/duplications) and/or methylation status in several types of disorders, including some that would otherwise remain undetected without prior knowledge of the molecular defect. Possible applications include the detection of common aneuploidies, microdeletions/microduplications in known syndromes, variants (and SNPs), subtelomere screening, characterisation of marker chromosomes, or determination of the methylation status of imprinted and promotor regions [methylation-sensitive MLPA (MS-MLPA)]. Commercial kits are available and are specific for each application thus the most appropriate kit should be used according to the referral reason (www.mrc-holland.com). Where commercial kits are used, manufacturer’s instructions should be followed when preparing samples for MLPA application, including the recommended DNA extraction method.

Different types of tissues may be used such as CVS, AF or foetal blood, as well as postnatal blood or buccal swab samples. Processing uncultured AF and CVS samples for MLPA analysis should follow the same procedures as described above (see section Sample preparation). All the samples in one assay should be prepared by the same method to ensure assay consistency and allow appropriate comparison between samples and normal controls. Blood staining in an AF sample should be noted as there may be MCC present. For CVS samples, analysis of mesoderm (digested villi) is recommended due to the possibility of CPM in the trophoblast.

When using MLPA for the first time, or when changing the DNA extraction method or instruments used, it is essential to perform internal validation of the MLPA technique. It is recommended that 16 DNA samples from healthy individuals are used for this validation and the standard deviation (SD) result of the experiment should be < 0.10 for all probes.

A negative, a positive and three normal controls must be included in each MLPA reaction. Peak areas in the electrophoretogram should be used, after standardisation, for evaluation of CN variation of specific genomic sequences in each sample. Peak quality and quantitative data should be assessed using specific software (e.g., PeakScanner, Coffalyser, GeneMapper, etc). Comparison of results should be performed between samples of the same assay to avoid intra-assay variation. For the reference samples, the SD of all probes should be ≤ 0.1 and dosage quotients (DQ) between 0.85 and 1.15. For the test samples, the SD of the reference probes should be ≤ 0.1 and the DQ between 0.85 and 1.15.

Evaluation of CN variation should be performed using peak areas in the electrophoretogram, after normalisation. The DQ obtained therein are used for the interpretation of results as follows: 0.85 < DQ < 1.15 indicates a normal result; 1.35 < DQ < 1.55 indicates a heterozygous duplication; 1.70 < DQ < 2.20 indicates a triplication; 0.35 < DQ < 0.65 indicates a heterozygous deletion; DQ = 0 indicates a homozygous deletion; all other DQ values indicate an inconclusive result [19, 20].

A trisomy for a particular chromosome should be considered if at least four out of eight probe ratios for a certain chromosome are ≥ 1.30 and the relative probe ratios for the remaining four probes are close to 1.30. Monosomy X should be considered if the probe ratios for X chromosome probes are within the range of those of normal males and Y signals are absent. If probe ratios for Y chromosome probes are within the range for those of normal males and X chromosome probes are ~2 times those of normal males 47,XXY should be considered. On the other hand, if probe ratios for Y chromosome probes are ~2 times those of normal males and X chromosome probes are within the range of those of normal male then 47,XYY should be considered. If the relative probe signals for X chromosome probes are ~2.5–3 times those of normal males and Y signals are absent 47,XXX should be considered. In cases where a partial chromosome gain has occurred, a duplication of a specific region will result in a ratio > 1.30 of two (or more) consecutive (and corresponding) probes. Additional studies should be used to confirm and interpret the result.

For prenatal samples, it is recommended that chromosomes 13, 18 and 21 are always tested. If there is no protocol in place for routine testing of sex chromosomes, it is recommended that one is available for samples referred with ultrasound anomalies suggestive of Turner syndrome.

In MS-MLPA, most probes detect the methylation of the first cytosine nucleotide in a single *Hha*I site within the sequence detected by the probe (GmCGC). If methylation is absent for this particular CpG-site, it does not necessarily mean that the whole CpG island is unmethylated. It also should be noted that methylation status may vary across tissues (e.g., it is not recommended to be used in CVS) and age groups. Determination of the methylation status (detection of methylated and unmethylated regions) of specific imprinted regions is performed simultaneously with CN detection by carrying out a digestion step with a methylation-sensitive endonuclease (*Hha*I) before the PCR reaction. Absence or decrease of peak signals will reflect the hybridisation of MS-MLPA probes that hybridise to unmethylated target sequences, digested by *Hha*I (therefore not amplified). This allows for the detection of CN evaluation. In contrast, the presence of peak signals result from the hybridisation of MS-MLPA probes to methylated sequences, undigested by *Hha*I and amplified. This allows for evaluation of methylation changes. To determine the methylation status of each MS-MLPA probe, the peak pattern of each digested DNA samples should be compared with the corresponding undigested sample. The methylation profile of a test sample is assessed by comparing the probe methylation percentages obtained on the test sample to the percentages of the reference samples. In order to ensure methylation analysis reliability, two criteria (additional to the ones described in the beginning of this section) should be met for all digested samples: the probe signal of the ‘digestion control probes’ should be 4% or less of the corresponding probe signals in the undigested reactions; and the DQ of the reference probes should be between 0.70 and 1.30. The comparison of undigested samples and normal controls for each probe allows for the CN evaluation that should be performed as described above for ‘regular’ MLPA analysis. It is essential to include reference (positive as well as negative controls) samples in each run to ensure assay consistency. Reliable MS-MLPA results should yield SD < 10% for each probe when testing normal samples.

Limitations of MLPA technology should be taken into account when opting for this type of methodology and they include:

- The test’s performance may be affected and compromised by factors such as impurities in the DNA sample, incomplete DNA denaturation, the use of insufficient or too much sample DNA, the use of insufficient or unsuitable reference samples, problems with capillary electrophoresis or a poor data normalisation procedure.
- Target nucleic acid sequence polymorphisms may result in impairment of probe hybridisation and amplification, leading to decreased fragment signals and consequent erroneous interpretation as a deletion. Sequence variants may also be undetectable unless occurring in target sites included in the probe kit/mix and/or in sites corresponding to probe ligation points.
- MLPA does not allow for the detection of XXX triploidy, mosaicism, MCC, chimeras nor the determination of zygosity in twin pregnancies, which leads to the possibility of discrepant/false-negative results.
  In cases with a clinical indication of triploidy where an apparent female diploid result is obtained, it should not be reported unless confirmed by another technique (e.g., QF-PCR). In cases where MCC is suspected (blood-stained AF or maternal decidua on CVS), as it is not possible to distinguish between maternal and foetal genotypes an alternative method (e.g., QF-PCR) is recommended. Consequently, for prenatal and solid tissue samples QF-PCR is considered a more appropriate technique.
- The presence of pseudomosaicism may not be detected by MLPA, which would potentially result in a misdiagnosis (false-positive or false-negative results).

- MLPA is also not able to detect any changes that lie outside the target sequence of the probes and it cannot detect balanced rearrangements. Even when no aberrations are detected, the possibility remains that biological changes in that gene or chromosomal region do exist but remain undetected.

Follow-up analysis (e.g., karyotype) should be undertaken following the detection of an aneuploidy to identify structural rearrangements and assess the recurrence risk. The same holds true for subtelomeric screening. Where further confirmatory analysis of the original material is not possible, parental karyotyping is recommended. For samples referred with ultrasound abnormalities, with a normal MLPA result, array CGH, SNP-based array or karyotype analysis is recommended. On the other hand, MLPA may also be used for confirmatory purposes following FISH or microarray studies. Where normal MLPA results are found in samples referred with a high-risk NIPT result, it is recommended that discrepancy of results is highlighted and a statement that the MLPA diagnostic result supersedes the NIPT screening result should be included in the report. Where CPM is a possible cause of the discrepant CVS result, ultrasound monitoring should be recommended on the report.

As with the other tests described, MLPA results should be reported using the latest ISCN and it is essential that the kit name is given in the report. When obtaining DQ results that repeatedly lie in the inconclusive range of values, the report should state as much that a final result is pending a subsequent analysis (e.g., array or karyotype).

### Microarray-based techniques

Genome-wide microarray-based analysis (array) is used to detect chromosomal imbalances at a significantly higher resolution than routine cytogenetic analysis. Because of its higher diagnostic yield, genome-wide array analysis is recommended as a first-tier diagnostic test for patients with ID, autism, neurodevelopmental disorders and/or congenital anomalies. In prenatal diagnosis, array analysis is recommended in the case of abnormal foetal structural ultrasound anomalies.

There are several designs and array platforms available containing at least 60,000 oligonucleotide probes onto which a mix of a differentially labelled test and reference DNA sample is hybridised. A diagnostic array platform should achieve a resolution of at least 400–600 kb for prenatal [21, 22] and 200–400 kb for postnatal referrals of developmental delay and congenital anomalies [23, 24]. bacterial artificial chromosome (BAC) array platforms are therefore no longer considered suitable for routine pre- and postnatal diagnostics. Many of the array platforms nowadays make use of SNP oligonucleotide probes, often in combination with the aforementioned oligonucleotide CN probes. Depending on the type and design of the array platform, one-labelled DNA sample (either test or reference) is hybridised onto a SNP-based array. The digital array data from each SNP-based array experiment can subsequently be selected to perform so-called ‘in silico’ array CGH analysis. With genome-wide SNP-based array analysis it is possible to not only determine the relative amount of numerous DNA targets to detect CNVs (as in Oligo arrays), but also to genotype each DNA target encompassing a SNP. The genotype information of the SNPs in a DNA sample enables the detection of CN neutral changes, absence of heterozygosity (AOH), and thereby, after suitable follow-up testing, the possible identification of recessive disease gene variants, mosaic aneuploidy or uniparental disomy (UPD). Patient–parent (trio) information analysis can be used to screen for the presence of any form of chromosomal UPD in the patient and/or to determine the parental origin of a de novo CNV. Moreover, the outcome of a genotype analysis (which can be performed with any combination of two or three array datasets) can also be used as a final quality control by ruling out potential sample mismatches due to false paternity or sample mix-up.

The laboratory should clearly state in their laboratory documents the theoretical resolution of their array platform, determined in large by the number of the probes on the array and the spacing between them. Because many of the probes are not spaced uniformly it is better, if possible and applicable, to refer to the ‘average’ resolution, differentiating between the targeted and the backbone resolution. Inherent to the structure of the human genome, this average resolution is not achieved for all regions, such as the repetitive sequences in the centromeric and heterochromatic regions. As a consequence, supernumerary marker chromosomes that do not contain euchromatic material will not be detected by array analysis.

The practical resolution of an array expresses the ability to detect aberrations of a given (minimum) size. The detection criteria (i.e., the minimum size and number of probes to call a CNV), as well as the reporting criteria (i.e., size and sort of a CNV, as well as its gene content) should also be included in the laboratory standard operating documents.

Microarrays may be applied for a number of indications and sample types. For postnatal indications, DNA is mostly obtained from EDTA blood, but other tissues such as buccal swab cells, Epstein-Barr virus (EBV)-transformed cells and (un)cultured fibroblasts may also be used. For prenatal array analysis, DNA is isolated from chorionic villus cells, amniocytes or foetal blood, either cultured or uncultured. Microarray analysis of DNA prepared from products of conception and foetal tissues is used to investigate the cause of miscarriage and foetal abnormality. The minimum concentration and amount of input DNA for array depends on the array platform used, but the DNA concentration is preferably ≥ 70 ng/µl.

Each array experiment should meet the laboratory’s defined minimally required quality criteria, such as SD of the intensity ratios, SNP-QC (quality of the SNP allele data), and 'waviness' or GC (nucleotides) metric [23, 25]. Determining the pathogenicity of CNVs relies heavily on the frequency information from healthy control cohorts and databases with previously reported clinically relevant CNVs. In addition to the host laboratories’ own datasets and national registries, there are several public databases and internet resources with genotype and phenotype information that can be used for array data interpretation [26].

In line with the classification of nucleotide variants, it is recommended to classify CNVs into five classes based on the degree of likelihood of pathogenicity, which for CNVs is predominantly based on the frequency differencesbetween the general population and affected individuals [26, 27]:

- Class 1: Benign;

- Class 2: Likely benign;

- Class 3: Uncertain clinical relevance (uncertain significance);

- Class 4: Likely pathogenic;

- Class 5: Pathogenic.

In order to correctly assess the clinical relevance of a CNV one needs to take into account the following inheritance models and other aspects [28, 29]:

- Dominant de novo or inherited (phenotype information of parents is crucial);

- Recessive; compound heterozygote or homozygous variant;

- X-linked;

- Imprinted genes;

- Mosaicism; in patient or parent;

- Two-hit CNV model;

- CNV in a non-genic region containing a regulatory sequence of a nearby disease gene;

- Genomic position of a CN gain; follow-up testing by region-specific FISH metaphase analysis may be helpful to determine whether the gain is due to a tandem duplication, an insertion elsewhere in the genome or a supernumerary marker.

Prenatally a CNV should be reported if it is consistent with the ultrasound findings as it will possibly affect the management of the pregnancy or a future individual or the family and is actionable. However, care should be taken when interpreting the identification of known pathogenic CNVs in a prenatal context in the absence of ultrasound anomalies as there is clinical ascertainment bias within the postnatal population [30].

The array results should be summarised in the latest ISCN and described in a clear and concise way, using correct terminology (dominant, recessive, heterozygous, homozygous, hemizygous, etc.). Class 1 and 2 variants are not to be included in the final report, unless, for example, it concerns a benign CN loss encompassing a recessive disease gene matching the clinical phenotype of the patient. One such example is a common 130-kb CN loss in 2q13 encompassing the *NPHP1* gene. Even though a heterozygous loss is frequently encountered in healthy individuals and hence classified as a (likely) benign CNV, a homozygous loss will cause nephronophthisis type I, a recessive cystic kidney disease (OMIM #256100). The accuracy of the CNV size(s) reported should be in line with the platform’s practical resolution. If appropriate, the CNV’s clinically relevant genes related to the referral reason and/or clinical features of the patient analysed are mentioned in the report. It is recommended to clearly state whether or not the reported finding could be causative for the clinical phenotype of the patient. In the case of a clearly defined associated phenotype, not only the respective gene(s) or established deletion/duplication region should be stated, but it is helpful to also include more information/references, i.e., the syndrome name (if applicable), respective OMIM number(s) and/or recent and relevant (review) papers for additional information. References may be added to the report, as long as these are not too old or generic, and should be cited in a format that allows the reader to easily identify the original article. A schematic representation of the aberrant region(s) and its gene content may be included for illustration. It is good practice to suggest suitable follow-up testing in patient and/or parents and clearly state, which samples are required for these specific test(s).

AOH should only be mentioned in the report if significantly increased from normal [31]:

- A single region of homozygosity (ROH) ≥ 10 Mb, which may be an indication for uniparental heterodisomy (UPD) or identity by descent (IBD);

- > 1% homozygous stretches on the autosomal genome (excluding X and Y), which may be an indication for parental consanguinity, hence an increased risk for a mutated, recessive disease gene.

It is recommended to only specify increased homozygosity in the ISCN description if it involves a single chromosome. If multiple chromosomes show significant homozygous stretches, mentioning it in the report would be more appropriate, e.g., a total of 145 Mb of homozygous stretches were detected (~4.8% of the autosomal genome). It should be noted that increased homozygosity can also be detected in unaffected individuals (but with a lower incidence) and is not in itself pathogenic, but adequate follow-up testing may identify the actual disease cause (i.e., UPD or a mutated recessive disease gene) if increased homozygosity is detected in a patient. The individual laboratory reporting criteria for AOH for both prenatal and postnatal referrals should also be clearly included in the laboratory standard operating documents.

Genome-wide array analysis cannot detect balanced rearrangements (including those that involve heterochromatin), small supernumerary marker chromosomes, low-level mosaicism or a nucleotide variant. Only SNP-based arrays can detect ploidy changes and UPD so when using array platforms with no or a limited number of SNPs these limitations should be included in the report.

### Next-generation sequencing (NGS) for CNV detection

NGS is a general term for a variety of methods and it has a number of applications, including NIPT for prenatal aneuploidy screening and whole-exome sequencing (WES), which are both used in a growing number of genome diagnostic laboratories. The targeted capture of exomes in combination with NGS based on short-read length technology (50–300 bp reads) and high-density coverage ( > 30 × ) of the captured fragments makes WES a promising, efficient and cost-effective approach for genetic testing. The focus on the exome (~50 Mb) is justified, because thus far, approximately 85% of disease-causing variants have been identified in exons or at splice-junction boundaries in introns. In addition to sequence variants, structural variants or CNVs can also be detected in WES data, which increases the diagnostic yield. Although widespread application is currently prohibited both by cost and the requirement for complex algorithms and data analysis pathways, it is predicted that this will become a standard approach for genetic diagnosis in coming years [32].

The interpretation of CNV in NGS data follows the same rules as mentioned under array-based techniques (see section Microarray-based techniques).

### Whole-exome sequencing

Each WES experiment should meet the laboratory’s defined and documented minimally required quality criteria, including base calling, mapping, coverage analysis and variant calling, as detailed in Weiss et al. [33], Aziz et al. [34] and Matthijs et al. [35].

Validation of a CNV in exome data by genome-wide array analysis or another suitable technique is highly recommended in case a finding is based on a limited number of aberrant targets and/or an unexpected finding is encountered, i.e., a large chromosomal imbalance in a person without ID or an apparently mosaic finding.

The analysis, interpretation and reporting of CNV in exome data should be in line with the referral reason, as well as with the informed consent – i.e., permission for gene panel (targeted) only or including ‘whole exome’. Relevant CNVs detected in exome data should be summarised in a cytogenomic description according to the latest ISCN. These relevant CNV in exome data results should be described in the report in a clear and concise way as mentioned under array-based techniques. The results from nucleotide variant and CNV analysis in exome data are obtained from a single experiment and must therefore be combined in a single report.

WES data analysis cannot detect balanced rearrangements or low-level mosaicism. Ploidy changes may only be detectable if suitable tools are employed in an adequate way and these limitations should be included in the exome report.

## Electronic supplementary material

Appendix A

Overview Video explaining why the guidelines are important

Tube - video
