# Supplementary material for: European guidelines for constitutional cytogenomic analysis
Source: Eur J Hum Genet. 2018 Oct 1;27(1):1–16. doi: 10.1038/s41431-018-0244-x (PMC6303289; doi:10.1038/s41431-018-0244-x)
Supplement: Supplementary file 1 — Appendix A [file 41431_2018_244_MOESM1_ESM.docx]

**APPENDIX A**

**National Guidelines**

AUSTRALIA

- Best Practice Guidelines for Chromosomal Microarray for Australian Laboratories. <https://www.hgsa.org.au/documents/item/6911>
- Requirements for cytogenetic testing : National Pathology Accreditation Advisory Council - Commonwealth of Australia, 2013 <http://www.health.gov.au/internet/main/publishing.nsf/Content/health-npaac-docs-CytogenTest.htm>.

BELGIUM

- Guidelines for Clinical Cytogenetic Diagnostic Laboratories in Belgium – Belgium Society of Human Genetics, 2004.
- Vanakker O, [Vilain](https://biblio.ugent.be/publication?q=author%3D%22Vilain%2C+Catheline*%22+or+(type+exact+bookEditor+and+editor%3D%22Vilain%2C+Catheline*%22)) C,  [Janssens](https://biblio.ugent.be/publication?q=author%3D%22Janssens%2C+Katrien*%22+or+(type+exact+bookEditor+and+editor%3D%22Janssens%2C+Katrien*%22)) K, [Van der Aa](https://biblio.ugent.be/publication?q=author%3D%22Van+der+Aa%2C+Nathalie*%22+or+(type+exact+bookEditor+and+editor%3D%22Van+der+Aa%2C+Nathalie*%22)) N, [Smits](https://biblio.ugent.be/publication?q=author%3D%22Smits%2C+Guillaume*%22+or+(type+exact+bookEditor+and+editor%3D%22Smits%2C+Guillaume*%22)) G,  [Bandelier](https://biblio.ugent.be/publication?q=author%3D%22Bandelier%2C+Claude*%22+or+(type+exact+bookEditor+and+editor%3D%22Bandelier%2C+Claude*%22)) C,  [Blaumeiser](https://biblio.ugent.be/publication?q=author%3D%22Blaumeiser%2C+Bettina*%22+or+(type+exact+bookEditor+and+editor%3D%22Blaumeiser%2C+Bettina*%22)) B, [Bulk](https://biblio.ugent.be/publication?q=author%3D%22Bulk%2C+Saskia*%22+or+(type+exact+bookEditor+and+editor%3D%22Bulk%2C+Saskia*%22)) S,  [Caberg](https://biblio.ugent.be/publication?q=author%3D%22Caberg%2C+Jean-Hubert*%22+or+(type+exact+bookEditor+and+editor%3D%22Caberg%2C+Jean-Hubert*%22)) J-H,  [De Leener](https://biblio.ugent.be/publication?q=author%3D%22De+Leener%2C+Anne*%22+or+(type+exact+bookEditor+and+editor%3D%22De+Leener%2C+Anne*%22)) A, et al. Implementation of genomic arrays in prenatal diagnosis: The Belgium approach to meet the challenges. Eur. J. Med. Genet. 2014; 57: 151-156

CANADA

- CCMG Cytogenetic Guidelines – Canadian College of Medical Genetics, 2010.

FRANCE

- Guide de Bonnes Pratiques en Cytogénétique – Asso­ciation des Cytogénéticiens de Langue Française, 2014.
- Guides des bonnes pratiques de l'analyse chromosomique sur puce a ADN (ACPA) 2013

GERMANY

- S2-Leitlinie Humangenetische Diagnostik. Deut­sche Gesellschaft fur Humangenetik e.V (GfH), Berufs­verband Deutscher Humangenetiker e.V (BVDH), 2011, medgen 23: 218-322.

ITALY

- Guidelines for cytogenetics of the Italian Society of Human Genetics 2013      www.sigu.net
- Recommendations for the Prenatal Use of Chromosomal Microarray Analysis SIGU – Italian Society of Human Genetics -SIEOG – Italian Society of Obstetric and Gynecologic Ultrasound and Biophysical Methods and   Volume 3, Number 1 2015  Perspectives Prenatal   Official Newsletter of the International Society for Prenatal Diagnosis  www.sigu.net

NETHERLANDS

- Richtlijnen constitutionele Cytogenetica (versie 1, 5-3-2015) http://vkgl.nl/nl/diagnostiek/formulieren-documenten-kwaliteit/category/7-veldnormen .
- Weiss MM1, Van der Zwaag B, Jongbloed JD, Vogel MJ, Brüggenwirth HT, Lekanne Deprez RH, Mook O, Ruivenkamp CA, van Slegtenhorst MA, van den Wijngaard A, Waisfisz Q, Nelen MR, van der Stoep N. Best practice guidelines for the use of next-generation sequencing applications in genome diagnostics: a national collaborative study of Dutch genome diagnostic laboratories. Hum Mutat. 2013;34:1313-21.

SWEDEN

- Riktlinjer för kvalitetssäkring i klinisk genetisk verksamhet 2011 <http://sfmg.se/download/riktlinjer/Kvalitetsriktlinjer/sfmg_riktlinjer-for-kvalitetssakring_rev101228.pdf>

UK

- ACC Professional Guidelines for Clinical Cyto­ge­netics: General Best Practice v1.04, 2007. <http://www.acgs.uk.com/committees/quality-committee/best-practice-guidelines/>
- ACC Professional Guidelines for Clinical Cyto­ge­netics: Prenatal Diagnosis v1.0, 2009. <http://www.acgs.uk.com/committees/quality-committee/best-practice-guidelines/>
- ACC: Professional Guidelines for Clinical Cyto­ge­netics: Constitutional postnatal chromosomal microarray best practice guidelines (2011) v2.00. <http://www.acgs.uk.com/committees/quality-committee/best-practice-guidelines/>
- ACC: Professional Guidelines for Clinical Cyto­ge­netics. Postnatal Best Practice Guidelines v1.01. March 2007. <http://www.acgs.uk.com/committees/quality-committee/best-practice-guidelines/>
- ACGS General Genetic Laboratory Reporting Recommendations (2015) <http://www.acgs.uk.com/committees/quality-committee/best-practice-guidelines/>
- ACGS QF-PCR for the diagnosis of aneuploidy best practice guidelines (2012) v3.01 <http://www.acgs.uk.com/committees/quality-committee/best-practice-guidelines/>
- Data Protection Act, 1998 & 2003
- HSE, Advisory Committee on Dangerous Pathogens (ACDP), The management and design and operation of microbial containment laboratories 2001 (ISBN 9780717620340)
- Royal College of Obstetricians and Gynaecologists. Green top guideline no.17. The investigation and treatment of couples with recurrent first trimester and second trimester miscarriages. April 2011.
- Royal College of Pathologists: Recommendations for the use of chromosome microarrays in pregnancy. 2015. <https://www.rcpath.org/resourceLibrary/recommendations-for-the-use-of-chromosome-microarray-in-pregnancy.html>
- Royal College of Pathologists: The Retention and Storage of Pathological Records and specimens (5^th^ edition), 2015.

UNITED STATES

- Standards and Guidelines for Clinical Genetics Labor­atories - American College of Medical Genetics, 2008.
- Aziz N, Zhao Q, Bry L, Driscoll DK, Funke B, Gibson JS, Grody WW, Hegde MR, Hoeltge GA, Leonard DG, Merker JD, Nagarajan R, Palicki LA, Robetorye RS, Schrijver I, Weck KE, Voelkerding KV. College of American Pathologists' laboratory standards for next-generation sequencing clinical tests. Arch Pathol Lab Med. 2015;139:481-93
- Kearney HM, South ST, Wolff DJ, Lamb A, Hamosh A, Rao KW; Working Group of the American College of Medical Genetics. American College of Medical Genetics recommendations for the design and performance expectations for clinical genomic copy number microarrays intended for use in the postnatal setting for detection of constitutional abnormalities. Genet Med. 2011;13:676-9.
- Kearney HM, Thorland EC, Brown KK, Quintero-Rivera F, South ST; Working Group of the American College of Medical Genetics Laboratory Quality Assurance Committee. American College of Medical Genetics standards and guidelines for interpretation and reporting of postnatal constitutional copy number variants. Genet Med. 2011; 13:680-5.
- Mascarello JT, Hirsch B, Kearney HM, Ketterling RP, Olson SB, Quigley DI, Rao KW, Tepperberg JH, Tsuchiya KD and Wiktor AE.  A Working Group of the American College of Medical Genetics (ACMG) Laboratory Quality Assurance Committee. Section E9 of the American College of Medical Genetics technical standards and guidelines: Fluorescence in situ hybridization *Genetics in Medicine* (2011) **13**, 667–675; doi:10.1097/GIM.0b013e3182227295
- Richards S, Aziz N, Bale S, Bick D, Das S, Gastier-Foster J, Grody WW, Hegde M, Lyon E, Spector E, Voelkerding K, Rehm H; ACMG Laboratory Quality Assurance Committee. Standards and guidelines for the interpretation of sequence variants: a joint consensus recommendation of the American College of Medical Genetics and Genomics and the Association for Molecular Pathology. Genet Med. 2015;17(5):405-24.
- South ST, Lee C, Lamb AN, Higgins AW, Kearney HM; Working Group for the American College of Medical Genetics and Genomics Laboratory Quality Assurance Committee. ACMG Standards and Guidelines for constitutional cytogenomic microarray analysis, including postnatal and prenatal applications: revision 2013. Genet Med. 2013; 15:901-9.

**International/European Standards**

- EU Directive Health and safety at work –Framework directive (89/391 EEC).
- EU Directive Health and safety at work - Carcinogens (90/394 EEC).
- EU Directive Health and safety at work – Manual Handling (90/269 EEC).
- EU Directive Health and safety at work – Work equipment (89/655)
- EU Directive Health and safety at work – Display screen Equipment (90/270 EEC)
- EU Directive Health and safety at work – Safety signs (92/58 EEC).
- EU Directive Health and safety at work – Pregnant workers (92/85 EEC).
- EU Directive Health and safety at work – Use of Protective equipment (89/656EEC).
- ISO 17025:2005: General requirements for the competence of testing and calibration laboratories.
- ISO 15189:2012. Medical Laboratories- particular requirements for quality and competence.
- ISO 9001: 2015 Quality Management systems requirements.
- ISO/IEC Guide 2: 2004 General terms and their definitions concerning standardization and related activity.
- Freedom of Information Act 2000.
- Data Protection Act 1998/2003. Convention for the protection of Human Rights and dignity of the human being with regard to the application of biology and medicine: convention on human rights and biomedicine, Oviedo, 1999.
- ISCN 2016: An International System for Human Cytogenomic Nomenclature (2016) Reprint of: Cytogenetic and Genome Research 2016, Vol. 149, No. 1-2. Editors: J. McGowan-Jordan, A. Simons, and M. Schmid.
- OECD Guidelines for Quality Assurance in Molecular Genetic testing (2007).
- E.C.A. General Guidelines: A common European framework for quality assessment for constitutional, acquired and molecular cytogenetic investigations. <http://www.e-c-a.eu/files/downloads/Guidelines/E.C.A._General_Guidelines_Version-2.0.pdf>
- Specific Constitutional Cytogenetic Guidelines. (2012) <http://www.e-c-a.eu/files/downloads/Guidelines/Specific_Constitutional_Guidelines_NL30.pdf>
- Matthijs G, Souche E, Alders M, Corveleyn A, Eck S, Feenstra I, Race V, Sistermans E, Sturm M, Weiss M, Yntema H, Bakker E, Scheffer H, Bauer P; EuroGentest; European Society of Human Genetics. Guidelines for diagnostic next-generation sequencing. Eur J Hum Genet. 2016;24:2-5.
