# Supplementary material for: European guidelines for constitutional cytogenomic analysis
Source: Eur J Hum Genet. 2018 Oct 1;27(1):1–16. doi: 10.1038/s41431-018-0244-x (PMC6303289; doi:10.1038/s41431-018-0244-x)
Supplement: Supplementary file 2 — Overview Video explaining why the guidelines are important [file 41431_2018_244_MOESM2_ESM.docx]

This video explains how the guidelines are a valuable tool for geneticists and can assist laboratories in the provision of a quality service.
